# Procedure of choice in a patient initially operated for a suspected epithelial appendiceal neoplasm

**DOI:** 10.1002/ccr3.4437

**Published:** 2021-07-10

**Authors:** Antigoni Xenou, Eugenia Vranou, Konstantinos A. Boulas, Maria Nathanailidou, Eytyxia Kyriakidou, Konstantinos Sitaridis, Isaac Filippidis, Anestis Hatzigeorgiadis

**Affiliations:** ^1^ Department of Radiology General Hospital of Drama Drama Greece; ^2^ Department of General Surgery General Hospital of Drama Drama Greece

**Keywords:** Appendix, appendiceal neoplasms, mucinous epithelial neoplasms, nonmucinous epithelial neoplasms, appendicectomy

## Abstract

In patients operated for a suspected appendiceal neoplasm, radical appendectomy is the procedure of choice because it provides definitive treatment in most of appendiceal neoplasms, except from mucinous or colonic‐type adenocarcinoma and NET>2 cm.

## Graphical abstract

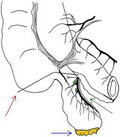

## 1 INTRODUCTION

**FIGURE 1:**
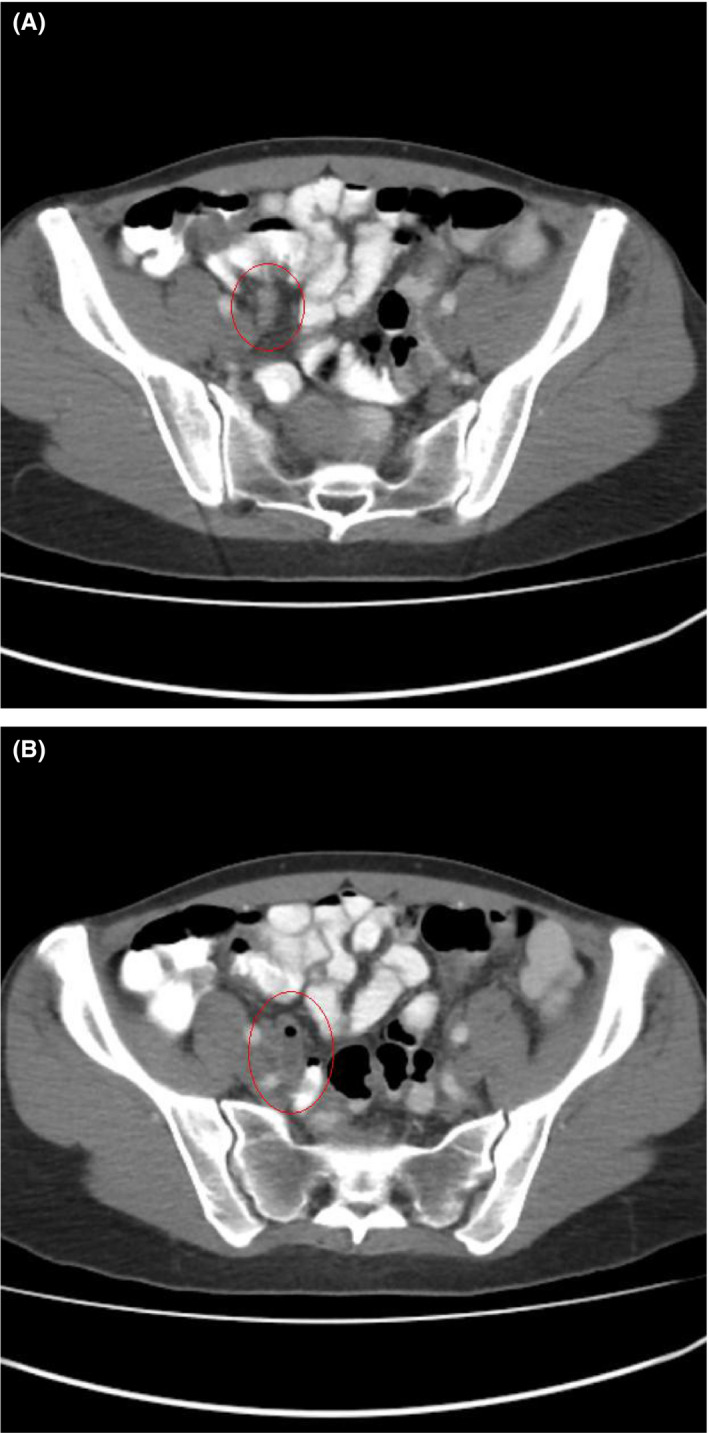
CT revealed 9‐mm wall thickening of the appendiceal base and body (Figure 1A) and 13‐mm dilatation of appendiceal tip with fluid accumulation (Figure 1B)

An otherwise healthy 43‐year‐old woman presented with a history of an unspecified lower abdominal pain over the last 6 weeks. Her body temperature was normal, and physical examination revealed no signs of peritoneal irritation. Complete blood count, CRP, CEA, CA19‐9, and CA125 were normal. CT revealed 9‐mm wall thickening of the appendiceal base and body (Figure 1A) and 13‐mm dilatation of the appendiceal tip with fluid accumulation (Figure 1B), absence of lymphadenopathy, and peritoneal involvement. Colonoscopy revealed a mass‐like protrusion at the appendiceal base without any synchronous colonic lesions. The patient was considered to have a suspected appendiceal neoplasm and scheduled for surgery.

## 2 QUIZ QUESTION: WHICH IS THE PROCEDURE OF CHOICE?

**FIGURE 2:**
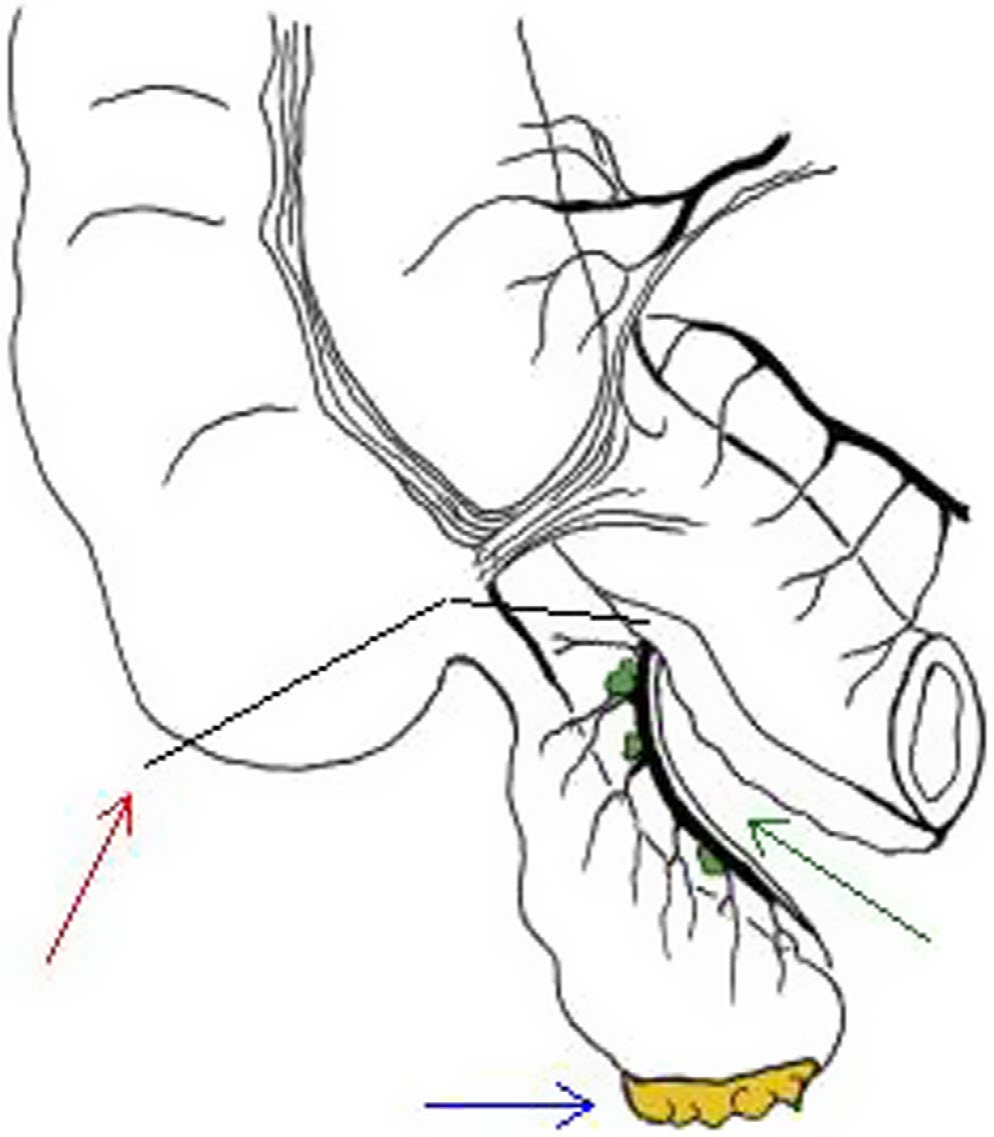
Radical appendectomy provides (1) negative resection margins at the appendiceal base through partial cecectomy (red arrow) and laterally by dissection through tissue planes; (2) valuable pathologic information by resection of periappendiceal peritoneum (blue arrow) and mesoappendix lymph nodes (green arrow)

Based on preoperative assessment, an inflammatory appendiceal process was ruled out. The most prominent diagnosis was a mucinous appendiceal neoplasm due to the mucocele‐like formation of the appendiceal tip. As histology was not known preoperatively, radical appendectomy with partial cecectomy (Figure 2) was performed, instead of simple appendectomy or right colectomy. In fact, surgical pathology reported a low‐grade appendiceal mucinous neoplasm resected in negative margins without appendiceal disruption and four negative mesoappendiceal lymph nodes. The postoperative period was uneventful.

In the present patient, radical appendectomy provided definitive treatment. In fact, radical appendectomy provided definitive treatment in most of the suspected appendiceal neoplasms (low‐ and high‐grade mucinous neoplasms, NET<2 cm, lymphoma), except from mucinous or colonic‐type adenocarcinoma which require right colectomy.^1^ Moreover, radical appendectomy should be the initial procedure of choice if pseudomyxoma peritonei was discovered intraoperatively, as upfront right colectomy does not provide any survival benefit if interval cytoreductive surgery is required.^2^

## CONFLICT OF INTEREST

The authors declare that they have no conflict of interests.

## AUTHOR CONTRIBUTIONS

All authors equally accessed the data and contributed to the preparation of the manuscript. BKA and HA were equally responsible for making and performing treatment decisions. HA reviewed the manuscript for critical intellectual content and approved the final version.

## STATEMENT OF HUMAN AND ANIMAL RIGHTS

The present article does not contain any studies with human or animal subjects performed by any of the authors.

## INFORMED CONSENT

Informed consent was obtained from the patient.

## Data Availability

Data sharing is not applicable to this article as no datasets were generated or analyzed during the current study.
